# Disease and Predation: Sorting out Causes of a Bighorn Sheep (*Ovis canadensis*) Decline

**DOI:** 10.1371/journal.pone.0088271

**Published:** 2014-02-07

**Authors:** Joshua B. Smith, Jonathan A. Jenks, Troy W. Grovenburg, Robert W. Klaver

**Affiliations:** 1 Department of Natural Resource Management, South Dakota State University, Brookings, South Dakota, United States of America; 2 Iowa Cooperative Fish and Wildlife Research Unit and Department of Natural Resource Ecology and Management, Iowa State University, Ames, Iowa, United States of America; BiK-F Biodiversity and Climate Research Center, Germany

## Abstract

Estimating survival and documenting causes and timing of mortality events in neonate bighorn sheep (*Ovis canadensis*) improves understanding of population ecology and factors influencing recruitment. During 2010–2012, we captured and radiocollared 74 neonates in the Black Hills, South Dakota, of which 95% (70) died before 52 weeks of age. Pneumonia (36%) was the leading cause of mortality followed by predation (30%). We used known fate analysis in Program MARK to estimate weekly survival rates and investigate the influence of intrinsic variables on 52-week survival. Model {S_1 wk, 2–8 wks, >8 wks_} had the lowest AIC*_c_* (Akaike’s Information Criterion corrected for small sample size) value, indicating that age (3-stage age-interval: 1 week, 2–8 weeks, and >8 weeks) best explained survival. Weekly survival estimates for 1 week, 2–8 weeks, and >8 weeks were 0.81 (95% CI = 0.70–0.88), 0.86 (95% CI = 0.81–0.90), and 0.94 (95% CI = 0.91–0.96), respectively. Overall probability of surviving 52 weeks was 0.02 (95% CI = 0.01–0.07). Of 70 documented mortalities, 21% occurred during the first week, 55% during weeks 2–8, and 23% occurred >8 weeks of age. We found pneumonia and predation were temporally heterogeneous with lambs most susceptible to predation during the first 2–3 weeks of life, while the greatest risk from pneumonia occurred from weeks 4–8. Our results indicated pneumonia was the major factor limiting recruitment followed by predation. Mortality from predation may have been partly compensatory to pneumonia and its effects were less pronounced as alternative prey became available. Given the high rates of pneumonia-caused mortality we observed, and the apparent lack of pneumonia-causing pathogens in bighorn populations in the western Black Hills, management activities should be geared towards eliminating contact between diseased and healthy populations.

## Introduction

Bighorn sheep (*Ovis canadensis*) populations in North America have declined dramatically since European settlement [1]. These declines have been attributed to an array of environmental and demographic factors including: unregulated hunting, predation, habitat loss, and diseases [2], [3]. While transplant efforts have proved effective in increasing overall bighorn numbers, many herds remain genetically and geographically isolated and often fail to recover to historical levels [4]. One of the major challenges currently facing managers attempting to restore these populations is low lamb recruitment.

In ungulates, juvenile survival is typically more variable than adult survival; thus, juvenile survival often has the greatest impact on population trajectories [5], [6]. While numerous studies have used vaginal implant transmitters (VITs) or intensely-monitored females to radiocollar and examine neonate survival of elk (*Cervus elaphus*, [7]) and deer (*Odocoileus* sp., [8], [9]), the steep and rugged terrain often used for lambing and rearing young [10] has precluded or severely limited this technique for neonate bighorn sheep [11]. Instead, most researchers have relied on visual observations of marked ewes for lambs at-heel, or lamb:ewe ratios in the herd [12]–[16]. Reliance on such metrics potentially allows reasonable assessments of overall recruitment into the population; however, it may obscure timing, causes of mortality, and may not reflect total mortality as such things as stillborn and early-age mortalities may be misconstrued as non-lambing events. Furthermore, it precludes the use of intrinsic variables (e.g., sex, weight) in survival analyses.

Documenting cause of mortality of juveniles is particularly important for bighorn sheep as many populations commonly experience pneumonia outbreaks that result in partial or complete die-offs [17], [18]. These die-offs are typically followed by years of depressed lamb recruitment that hinder population recovery. Additionally, cougar (*Puma concolor*) predation on adults has been shown to contribute to some bighorn sheep population declines [19]–[22] with higher rates of predation occurring during declines in primary prey [23]. Predation by cougars also was the suspected cause of reduced lamb survival in the eastern Black Hills [24].

As in many other regions of the United States [25], native bighorn sheep were extirpated from the Black Hills, South Dakota, around the early 1900 s [24] and western South Dakota around 1925 [26]. Reintroductions and transplants beginning in 1965 resulted in the establishment of 5 subherds in the Black Hills region. Beginning in 2006, surveys conducted annually by South Dakota Department of Game, Fish and Parks (SDGF&P) indicated significant declines in bighorn lamb recruitment in 3 subherds (i.e., Rapid Creek, Hill City, and Spring Creek) in the east-central Black Hills (SDGF&P, Rapid City, SD, unpublished data). Our objectives were to radiocollar neonate bighorn sheep to: 1) estimate survival and document cause-specific mortality of bighorn lambs in the eastern Black Hills, South Dakota and 2) determine the influence of intrinsic variables on neonate survival.

## Materials and Methods

### Study Area

The Black Hills are located in southwestern South Dakota and eastern Wyoming, USA. Topography of the area varied from steep ridges, rock outcrops, canyonlands, and gulches to upland prairie, rolling hills, and tablelands. Elevations ranged from 973 to 2,202 m above mean sea level (msl; [27]). Ponderosa pine (*Pinus ponderosa*) forest comprised 83% of the landscape [28]. Mixed grass prairie (5%), riparian (4%), aspen (*Populus tremuloides*)-mixed conifer forest (3%), and developed open space (2%) were other major land cover types present in our study area [28]. During our study, average annual precipitation in the project area was 53 cm. Mean temperatures ranged from a maximum of 28°C in July to a minimum of −10°C in January. Climate values were based on data collected at the Hill City, South Dakota weather station from 1981–2010 [29].

**Figure 1:**
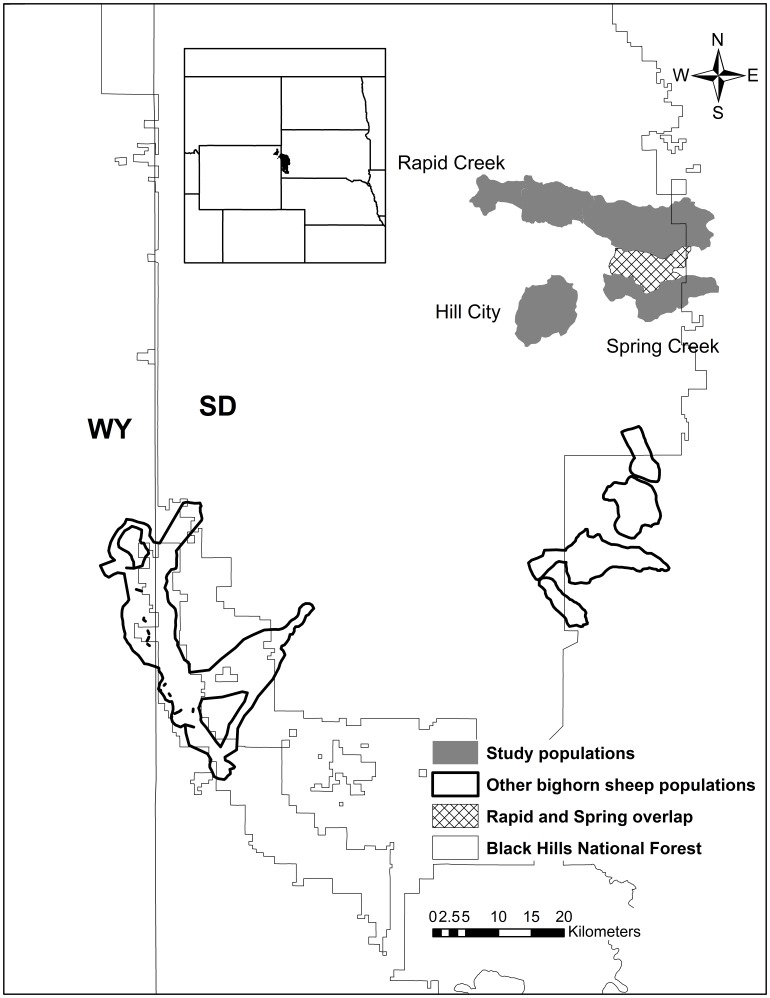
Bighorn sheep populations and locations of study populations in the Black Hills, South Dakota, USA, 2010–2012.

The study area for this project was located in the east-central portion of the Black Hills with bighorn sheep habitat encompassing an area of approximately 26,000 ha. Each herd maintained distinct wintering areas; however, we did observe some range overlap between Spring Creek and Rapid Creek ewes during the lambing season (Figure 1). Over the course of our study, no range overlap was observed between our study herds and that of other herds in the Black Hills. In 2010, breeding-age ewe population estimates were: Rapid Creek = 56, Spring Creek = 50, and Hill City = 10 (SDGF&P, Rapid City, SD, unpublished data). Estimated proportion of ewes collared by herd across years ranged from: Rapid Creek 25%–29% (2010–2012), Spring Creek 30%–42% (2010–2012), and Hill City 90%–100% (2011–2012). Previously, no all-age pneumonia outbreaks had been detected in these herds, although several lambs had tested positive for pneumonia prior to 2010 (S. Griffin, SDGF&P, Rapid City, SD, personal communication). There were no domestic sheep grazing allotments within the Black Hills National Forest; however, several small domestic sheep and goat flocks were kept on private lands within bighorn sheep use areas. Other ungulates in the study area included mule deer (*Odocoileus hemionus*), white-tailed deer (*O. virginianus*), mountain goats (*Oreamnos americanus*), and elk. In addition to cougars, other potential predators of bighorn sheep included coyotes (*Canis latrans*) and bobcats (*Lynx rufus*).

### Ewe Capture

We captured adult ewes using a drop-net baited with weed-free alfalfa hay or sheep were chemically immobilized (BAM; 0.43 mg/kg butorphanol, 0.29 mg/kg azaperone, 0.17 mg/kg medetomidine) via dart rifle (Dan-Inject, Børkop, Denmark, EU). We estimated ewe age class (1 year, 2 years, 3 years, or ≥4 years) based on tooth replacement [30]. We evaluated pregnancy status of ewes via ultrasonography (Universal Ultrasound, Bedford Hills, NY, USA) at time of capture. We fitted pregnant ewes with M3930 VITs manufactured by Advanced Telemetry Systems (ATS; Isanti, MN) with a redesigned wing system and antenna length of 6 cm [31]. Ewes that were not pregnant or not checked for pregnancy at the time of capture were not fitted with VITs. Methods of VIT deployment followed Bishop et al. [31]. In addition to receiving VITs, all ewes were fitted with very high frequency (VHF) collars (M252OB or G2110D; ATS) that were uniquely marked to facilitate individual identification.

### Lamb Capture Using Ewes with VITs

Prior to the lambing season, radiocollared ewes were monitored 1–3 times per week from the ground using hand-held directional antennas (Telonics, Inc., Mesa, AZ), or from a Cessna 182 airplane. We listened for possible VIT expulsion each time we located ewes. When we detected an expelled VIT prior to the lambing season, we retrieved it using ground telemetry, ascertained if the ewe had aborted the fetus on-site, and estimated date of expulsion as the mean date between the first mortality signal and the last active signal received.

During the lambing season in May and June, ewes with VITs were checked once daily to determine if the VIT had been expelled. If the radio signal indicated a VIT had been expelled and terrain permitted, personnel would use telemetry to locate the expelled VIT on foot and retrieve it. If the VIT was located at a birth site and the lamb was present, we attempted to hand-capture it. If the dam had moved away from the VIT or if a lamb was not located in the vicinity of the ewe, we searched the area surrounding the ewe’s location and the VIT location, and if a lamb was located we attempted capture. In the event the VIT was prematurely expelled based on a lack of evidence of birthing activities at the VIT site and observation of the ewe without a lamb, we intensively monitored the individual ewe’s behavior. If we subsequently established the ewe had lambed, we attempted to capture the lamb once it was observed.

### Lamb Capture Using Ewes without VITs

We monitored radiocollared ewes without VITs on a near daily basis for movement patterns indicative of parturition and presence of newborn lambs via radio-telemetry and visual observation from a distance. When we detected a newborn lamb, we assessed its degree of mobility using observations of ambulatory movements. We attempted hand-capture from the ground if the lamb seemed sufficiently immobile and the terrain was accessible. We waited until animals bedded down before attempting capture. Solitary ewe-lamb pairs were preferred; however, we also attempted captures of lambs associated with small groups of ewes. Once animals bedded down, we noted location of the animals in relation to topography and notable landmarks. Ideally, while attempting to avoid detection (e.g., by climbing up the opposite side of a ridge), two people approached the animals from above. When detection by the animals was imminent, we rapidly approached the animals’ location causing the ewe to flee, and the lamb would hide or attempt to flee at which time we attempted to capture the lamb.

### Lamb Handling and Marking

We physically restrained each captured lamb, blindfolded, and fitted the lamb with an expandable, 62 g VHF collar equipped with a 4-hr or 8-hr mortality switch (Model M4210; ATS). Additionally, we determined sex, age, and weight of captured lambs. We monitored lamb survival after capture using telemetry to determine if lambs had died or were abandoned as a result of our capture activities. We attempted to keep handling time to <5 minutes. All capture and handling procedures were approved by the South Dakota State University Animal Care and Use Committee (Approval number 09–019A) and followed recommendations of the American Society of Mammalogists [32].

We monitored lambs and ewes daily for 60 days post-capture and 3–4 times/week thereafter from the ground using a receiver and hand-held directional antenna (Telonics, Inc.) or from a Cessna 182 airplane. When we detected a mortality signal, we immediately located the collar, and recorded evidence at the site of mortality to determine cause of death. If we could not determine cause of death in the field, we transported animals to the Washington Animal Disease Diagnostic Laboratory (WADDL) at Washington State University for further examination. We classified mortalities as predation based on observations at the mortality site including, bite marks, caching, plucking, blood, and consumption of carcass. To estimate survival and determine factors influencing lamb survival, we used the known fate model with the logit-link function in Program MARK [33]. We estimated weekly survival for 52 weeks post capture. Intrinsic variables included capture year, sex, herd, mass at capture, age at capture, winter severity, cougar population estimate for the Black Hills, birth timing (early, peak, and late), and 4 age-intervals (Table 1).

**Table 1:** A priori models constucted to determine the influence of intrinsic variables on bighorn sheep neonate survival in the Black Hills, South Dakota, USA, 2010–2012.

Model	K^a^	Description
S_constant_	1	Survival was constant
S_vit status_	2	Survival varied by whether ewe was vitted or non-vitted
S_age at capt_	2	Survival varied by age at capture of neonates
S_weight_	2	Survival varied by birth weight of neonates
S_birth timing_ b	3	Survival varied by birth timing (early, late, and peak)
S_year_	3	Survival varied by year
S_winter severity_	2	Survival varied by previous winter severity
S_cougar pop_	2	Survival varied by estimated cougar population
S_herd_	3	Survival varied by herd
S_sex_	2	Survival varied by gender
S_1 wk, >1 wk_	2	Survival varied by age in 2 stages
S_1 wk,2–4 wks, >4 wks_	3	Survival varied by age in 3 stages
S_1 wk, 2–8 wks, >8 wks_	3	Survival varied by age in 3 stages
S_1 wk,2–4 wks, 5–8 wks, > 8 wks_	4	Survival varied by age in 4 stages

a Number of parameters.

b Peak = date when 50% of known lambs were born +/−3 days, early = born >3 days before peak parturition date, and late = born >3 days after peak parturition date.

### Survival Analysis

We determined age of the lamb at capture on the basis of new hoof growth measurements and texture, umbilicus condition, behavioral characteristics such as mobility, the presence of afterbirth, and wet fur. We calculated winter severity by summing days with measurable snow accumulation with days that were ≤−7°C based on data obtained from Hill City (for Spring Creek and Hill City herds) and Rapid City (for Rapid Creek herd), South Dakota weather stations from 2009–2012 [29]. Cougar population estimates were based on mark/recapture and modeling of the Black Hills cougar population (J. A. Jenks, South Dakota State University, Brookings, SD, unpublished data). Stage-interval models were constructed to test hypotheses regarding lamb susceptibility to various sources of mortality (e.g., predation vs. pneumonia). For birth timing, we grouped neonates into 3 periods: peak born (date when 50% of known lambs were born ±3 days), early (born >3 days before peak parturition date), and late (born >3 days after peak parturition date). We also considered 4 age-intervals: 1) a 2-stage model (S_1wk, >1 wk_) in which neonate survival varied from <1 week versus >1 week post birth, 2) a 3-stage model (S_1 wk,2–4 wks, >4 wks_) in which neonate survival varied among 1 week, 2–4 weeks, and >4 weeks post birth, 3) a 3-stage model (S_1 wk, 2–8 wks, >8 wks_) in which neonate survival varied among 1 week, 2–8 weeks, and >8 weeks post birth, and 4) a 4-stage model (S_1 wk, 2–4 wks, 5–8 wks, >8 wks_) in which neonate survival varied among 1 week, 2–4 weeks, 5–8 weeks, and >8 weeks post birth (Table 1).

We based a priori model construction on variables we considered biologically meaningful to neonate ecology and used Akaike’s Information Criterion corrected for small sample size (AIC*_c_*) to select models that best described the data. We compared AIC*_c_* values to select the most parsimonious model and considered models differing by ≤2 ΔAIC*_c_* from the selected model as potential alternatives [34]. We used Akaike weights (*w_i_*) as an indication of support for each model. Because there is no current goodness-of-fit test statistic available for known fate models, we investigated model robustness by artificially inflating *ĉ* (i.e., a model term representing overdispersion) from 1.0 to 3.0 (i.e., no dispersion to extreme dispersion) to simulate various levels of dispersion reflected in Quasi-AIC*_c_* (QAIC*_c_*; [7], [35]). Additionally, as some lambs were collared from the same ewe over multiple years, we performed a data-bootstrap analysis [36] in Program MARK to estimate overdispersion as a function of lamb maternity. Our bootstrap analysis was performed on our top ranked survival model and comprised 10,000 replicate datasets generated by resampling our data with replacement after removing lambs associated with each ewe across years.

We calculated a cumulative incidence function (CIF) to estimate cause-specific mortality related to pneumonia and predation to measure the contribution of each to survival rates [37]. We used the *wild 1* package [38] in Program R to calculate CIF for all individuals that survived ≥1 day. We used a log-rank test to evaluate whether observed differences between cumulative mortality curves differed between the 2 mortality factors using the *survival* package [39] in Program R. The test computes a χ^2^ statistic for observed and expected mortality events during each time step and tests the null hypothesis of no difference between mortality curves.

## Results

From February 2010 to April 2012, we captured and radiocollared 55 adult ewes (3 at 3 years of age; 52 at ≥4 years of age) and deployed 62 VITs [11]. From May 2010 to June 2012, we captured and radiocollared 77 neonates (25 in 2010, 25 in 2011, and 27 in 2012), 2 of which were from unmarked ewes (lamb capture by ewe VIT status summarized in Smith et al. [11]). Peak parturition occurred on 13 May 2010 (range = 2–31 May), 16 May 2011 (range = 4–26 May), and 16 May 2012 (range = 30 April–6 June). Of the 77 neonates radiocollared, 14 (18.2%) were born early, 40 (51.9%) were born during the peak period, and 23 (29.9%) were born late. Estimated age at capture ranged from <0.01 to 2 days and 54% of lambs were <1 day old at capture; mean age and weight at capture was 0.8 days (SE = 0.1, *n* = 70) and 4.7 kg (SE = 0.1, *n* = 75), respectively. We documented 72 mortalities from capture to 52 weeks post capture; 24 in 2010, 23 in 2011, and 25 in 2012. However, in 2012, 2 lambs died from possible capture-related activity and 1 lamb was transported to a captive facility following possible capture-related abandonment; thus, we censored them from survival analyses. Additionally, 1 lamb in 2010 was right-censored 163 days post capture after we determined the collar was no longer on the animal. In addition to the 70 mortalities of radiocollared lambs, we documented 5 mortalities of lambs <24 hrs old; 3 stillborn, 1 predation, and 1 hypothermia. Because they were not collared, these animals also were excluded from survival analyses. Mean age at death was 42 days (SE = 5, *n* = 70).

From model results on survival analysis, we considered {S_1 wk, 2–8 wks, >8 wks_} as the best approximating model (*w_i_* = 0.59). Remaining models were ≥2.00 ΔAIC*_c_* units from this model, and the weight of evidence supporting this model was 1.4 times greater than all other models combined (Table 3). While 2 models, {S_1 wk,2–4 wks, 5–8 wks, >8 wks_} and {S_birth timing_}, were within ≤2.73 ΔAIC_c_ units from our top model, we excluded them for the following reasons; 1) survival estimates for weeks 2–4 (0.86, SE = 0.03) vs weeks 5–8 (0.86, SE = 0.03) from model {S_1 wk,2–4 wks, 5–8 wks, >8 wks_} were not significantly different and were virtually identical to the 2–8 week survival estimate (0.86, SE = 0.02) obtained from model {S_1 wk, 2–8 wks, >8 wks_}, 2) given the lack of discrepancy between these 2 models, removal of model {S_1 wk, 2–8 wks, >8 wks_} resulted in weight of evidence supporting our top ranked model (*w_i_* = 0.73) 2.7 times greater than all other models combined, and 3) the model {S_birth timing_} 95% CI for the β estimate for early born lambs incorporated 0. Furthermore, model {S_1 wk, 2–8 wks, >8 wks_} had the lowest QAIC*_c_* when *ĉ* = 2.0 (moderate dispersion; QAIC*_c_* wt = 0.34) and through *ĉ = *3.0 (extreme dispersion; QAIC*_c_* wt = 0.20). The β estimate and 95% confidence intervals for the intercept (default >8 weeks survival period; 2.78, 95% CI = 2.28 to 3.29), 1 week (−1.36, 95% CI = −2.11–−0.60), and 2–8 weeks age intervals (−0.96, 95% CI = −1.57–−0.36), indicated β ≠ 0; thus, we considered survival was best explained by 3-stage age-intervals. Weekly survival estimates for 1 week, 2–8 weeks, and >8 weeks were 0.81 (95% CI = 0.70–0.88), 0.86 (95% CI = 0.81–0.90), and 0.94 (95% CI = 0.91–0.96), respectively; overall probability of surviving 52 weeks was 0.02 (95% CI = 0.01–0.07). Of 70 mortalities used in covariate models, 15 (21.4%) occurred during the first week, 39 (55.7%) during weeks 2–8, and 16 (22.9%) occurred >8 weeks of age. Results of data bootstrapping analyses provided limited evidence for overdispersion (i.e., limited sibling dependence) due to lambs being collared from the same female over multiple years (*ĉ = *1.23). Our estimate of *ĉ* indicates sample variance was slightly underestimated; however, as we observed no change in our top ranked survival model after inflating *ĉ* to 3.0, we believe multiple births from some ewes had little impact on our overall estimate of survival.

**Figure 2:**
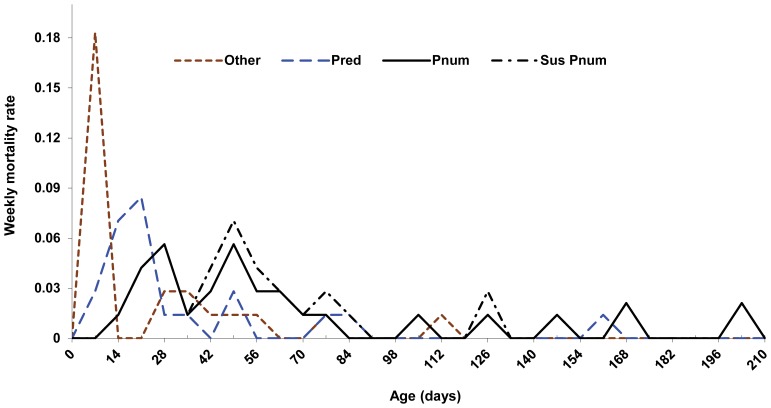
Average weekly mortality rate comparison of bighorn lamb mortality events. Average weekly mortality rate comparison for other^a^, predation, pneumonia, and suspected pneumonia^b^ mortality events of bighorn lambs in the Black Hills, South Dakota, USA, 2010–2012. ^a^ Other includes all causes of mortality other than predation and pneumonia. ^b^ Suspected pneumonia includes mortalities in which we assumed pneumonia was the ultimate or proximate cause of death in addition to confirmed pneumonia mortality events.

Pneumonia was the leading cause of mortality (35.7%, *n* = 25) followed by predation (30.0%, *n* = 21); we were unable to determine ultimate cause of death for 7 (10%) mortalities (Table 2). We verified cougar predation in 15 (71%) predation events, and suspected felid (cougar or bobcat) on 5 (24%) other occasions; canid (coyote or domestic dog [*C. lupus familiaris*]) was suspected in 1 (5%) instance. Additionally, we suspected pneumonia as the ultimate (6 unknowns) or proximate cause of death (1 predation event) in 7 other instances. In 6 cases, carcasses were too degraded for definitive diagnosis; however, carcasses were intact (i.e., no evidence of predation) and the mortalities occurred during peak times when lambs were most susceptible to the disease (Figure 2). Additionally, in one cougar-killed lamb we obtained sufficient tissue for analysis and pneumonia was detected.

**Table 2:** Cause-specific mortality of neonate bighorn sheep in the Black Hills, South Dakota, 2010–2012.

Cause-specific mortality	*n*	%
Pneumonia	25	35.7%
Predation	21	30.0%
Starved	8	11.4%
Unknown	7	10.1%
Ewe died	3	4.3%
Abandoned	1	1.4%
Contagious eczema (CE)	1	1.4%
Hypothermia	1	1.4%
Infection	1	1.4%
Underweight	1	1.4%
Vehicle	1	1.4%

**Table 3:** Top-ranked survival models of neonate bighorn sheep from birth to 52 weeks post capture in the Black Hills, South Dakota, USA, 2010–2012 when ĉ (a model term representing overdispersion) was 1.0 (i.e., assumed no dispersion).

Model^a^	AIC_c_ b	ΔAIC_c_ c	*w_i_* d	*K* e	Deviance
{S_1 wk, 2–8 wks, >8 wks_}	429.67	0.00	0.59	3	423.64
{S_1 wk,2–4 wks,_ _5–8 wks, >8 wks_}	431.70	2.02	0.36	4	423.63
{S_birth timing_}	432.40	2.73	0.26	3	426.36
{S_1 wk,2–4 wks, >4 wks_}	436.25	6.58	0.02	3	430.22

a Composition and description of models are listed in Table 1.

b Akaike’s Information Criterion corrected for small sample size (Burnham and Anderson 2002).

c Difference in AIC_c_ relative to min. AIC.

d Akaike wt (Burnham and Anderson 2002).

e Number of parameters.

The mortality curve from pneumonia was significantly different from predation (*X* ^2^ = 4.56, *df* = 1, *P* = 0.04), with average age of lambs succumbing to predation (35.5 days, SE = 8.9 days; median = 17.5 days) younger in age than those succumbing to pneumonia (60.3 days, SE = 9.8 days; median = 48.0 days). Risk of predation peaked around 21 days of age while pneumonia exhibited 2 peak periods, 28 and 49 days, before tapering off around day 84 (Figure 2). The CIF indicated the risk of mortality from predation (0.45, 95% CI = 0.30–0.58) was higher than for pneumonia (0.14, 95% CI = 0.02–0.25) during the first 21 days of life, while pneumonia (0.54, 95% CI = 0.39–0.68) was higher than predation (0.20, 95% CI = 0.05–0.34) for lambs surviving >21 days. Overall CIF for pneumonia and predation were 0.37 (95% CI = 0.25–0.48) and 0.30 (95% CI = 0.17–0.42), respectively.

## Discussion

Nearly all lambs in the herds we studied died in their first year, and all but one died by the age of 2. Of 82 documented birthing events only 3 (4%) lambs survived to 1 year of age (2 in 2011 and 1 in 2012). However, both surviving lambs from 2011 ultimately died the following year; one was struck by a vehicle while migrating back to the lambing grounds at just over 1 year of age and the other was found dead of unknown causes at approximately 16 months of age. Based on our sample of 74 collared animals, recruitment averaged 0.04 (SD = 0.04) across years (2010 = 0.00, 2011 = 0.08, 2012 = 0.04) and was lower than previous regional estimates (range = 0.10–0.28; 2007–2009; SDGF&P, Rapid City, SD, unpublished data) but was within the range of recruitment observed in 9 populations of bighorn sheep in the Hells Canyon area of Idaho, Oregon, and Washington that displayed evidence of pneumonia epizootics (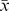 = 0.17, SD = 0.11, range 0.39–0.00; [15]).

Similar to our study, Cassirer and Sinclair [15] determined that pneumonia (86%) was the leading known cause of lamb mortality. However, they relied on visual observations and documented only 1 (4%) predation event. Based on our observations, ewes that lost lambs as a result of predation were more likely to leave the area where the predation event occurred, while ewes that lost lambs as a result of other mortality events (e.g., pneumonia, starvation) were more likely to remain in the general vicinity. When attempting to retrieve lambs that died from causes other than predation, we routinely observed ewes in the same area as the recently deceased lamb; however, on only one occasion did we observe a ewe within sight of a lamb that was killed by a predator. As a consequence of observed ewe behavior, relying on visual observations would have led to an underestimate of mortality from predation. Furthermore, we documented 5 lamb mortalities prior to capture (e.g., they died ≤24 hrs old), and had we been relying solely on visual observations these events would most likely have been misconstrued as non-lambing events resulting in a lower assessment of overall lamb mortality. It is worth noting that despite having numerous ewes instrumented with VITs and attempting to obtain visual observations on ewes not instrumented with VITs on a near daily basis, we observed several instances where ewes had apparently given birth (e.g., presence of afterbirth on the animal) yet we were unable to find the lamb. Although of minimal importance in our study, with higher survival, these mortalities could contribute significantly to total estimates of survival.

Model selection results indicated that neonate survival was best explained by 3-stage age-intervals. Previous research examining neonate survival in deer [9] and elk [7] have identified similar 3-phase models as best explaining survival. However, their results were mainly attributed to different predator avoidance strategies (e.g., hiding vs. fleeing; [40]) typically exhibited in these species. Rather than a difference in life-history phases, we believe our results were more a reflection of the different mortality sources acting at distinct time periods on these populations. For instance, during the first week of life lambs were most likely to die of causes other than predation or pneumonia (e.g., handling, starvation, infection), while during the second and third weeks of life lambs experienced the greatest risk of mortality from predation, and at >3 weeks pneumonia was the leading cause of mortality (Figure 2). Gaillard et al. [5] noted that preweaning juvenile mortality typically occurs within 1 month of birth, yet, due to the presence of pneumonia, we observed no difference in survival from 2–8 weeks of life.

Summer pneumonia epizootics resulting in high rates of lamb mortality followed a similar pattern to those documented in other populations [15], [41], with relatively few deaths occurring in the first few weeks. Lambs as young as 11 days died from pneumonia although the majority occurred ≥4 weeks of age (Figure 2). Cassirer and Sinclair [15] found that highest rates of pneumonia-related mortality occurred between 6–8 weeks post birth and suggested that morbidity may have coincided with the waning of passive immunity acquired from colostrum [42]. We found pneumonia-related mortality occurred slightly earlier, peaking from 4–7 weeks; however, we observed a definitive lull in mortality around week 5 (Figure 2). Lack of mortality at that time may simply be a result of sample size, or perhaps a function of the vigor in which the epizootic operated within each of the 3 herds. We did, however, find that birth weights of lambs that died of pneumonia ≤35 days old, were on average lighter (4.23 kg, SE = 0.14; *n* = 9) than lambs that died of pneumonia >35 days old (4.97 kg, SE = 0.10; *n* = 14) suggesting that heavier lambs lived longer.

Predation was our second leading cause of mortality with the greatest risk occurring primarily around 2–3 weeks of age. It is likely that decreased mobility during this time predisposed lambs to predation, although we suspect that changes in prey density also may explain some of the decreased risk at >3 weeks. For instance, birth peak for bighorn sheep was approximately 15 May across years, while the birth peak in the Black Hills for mule deer was 7–14 June [43], for white-tailed deer it was 7–17 June [44], and for elk it was 28 May–4 June (Schmitz 2010, SDGF&P, unpublished data). If risk of predation was strictly a function of lamb mobility we would expect no difference in predation based on birth timing (e.g., early, peak, or late). However, if predation was a function of prey density we would expect a decrease in risk from early to late born lambs as other prey became available. Early, peak, and late born lambs represented 18% (*n* = 14), 52% (*n* = 40), and 30% (*n* = 23), respectively, of all documented mortality events; yet, they made up 29% (*n* = 6), 62% (*n* = 13), and 10% (*n* = 2), respectively, of predation events. The decreasing trend in relative predation risk we observed between birth periods, and the decreased susceptibility to predation after 3 weeks of life, indicates that prey density could influence neonate lamb risk of predation, and supports others (e.g., [45]) who have hypothesized cougar predation on bighorn sheep is reduced when primary prey (deer; *Odocoileus spp*.) are more abundant.

Cassirer and Sinclair [15] noted a lack of lesions in predator-killed animals, no interaction between predation and disease-related mortality, and suggested that disease did not increase adult sheep vulnerability to predation. We, however, had evidence to the contrary in lambs. Although most predation events resulted in nearly the entire carcass being consumed, we were able to test one lamb that died at 81 days of age, and a second uncollared lamb that was found in the same cache pile. Both lambs were killed by a cougar the night before and laboratory (WADDL) results confirmed both had lesions consistent with bronchopneumonia. This was the only time we documented 2 lambs killed on the same evening by the same predator, and the fact that both were pneumonia positive suggests that disease can increase lamb vulnerability to predation. Additionally, the one lamb that died as a result of canid predation occurred when the lamb was approximately 158 days of age which, we assume, would have been sufficiently mobile to avoid canid predators had it been healthy. Yet, this lamb was observed 3 days prior to the mortality event and appeared gaunt and lethargic. Studies of domestic calves (*Bos taurus)* have indicated animals inoculated with *Mannheimia haemolytica* (one of the pathogens hypothesized to cause pneumonia in bighorn sheep) spent less time feeding and more time resting than control animals [46]. If these same behavioral characteristics were exhibited in bighorn lambs they would likely experience greater risk to predation.

Even though we considered only one model as best approximating survival, we did glean information from other models that was noteworthy. First, model {S_age at capt_} 95% CI for the β estimate incorporated 0 (−0.16, 95% CI = −0.62–0.31) and the estimate suggested no positive relationship between age at capture and survival. Based on these results, it did not seem that capturing younger lambs during the first few hours of life, a time we hypothesized may be a critical bonding period, influenced survival. Additionally, model {S_birth timing_} indicated that peak (0.90, 95% CI = 0.86–0.92) and early (0.93, 95% CI = 0.87–0.96) born lambs exhibited higher survival than late (0.78, 95% CI = 0.66–0.86) born lambs. As noted above, late born lambs were less likely to suffer mortality from predation, however, the opposite trend was observed for late born lambs dying of pneumonia. Late born lambs were 1.5 times (11 observed vs 7.1 expected) more likely to die of pneumonia than expected by chance, which was higher than for early (1.2; 6 observed vs 5 expected) or peak (0.6; 8 observed vs 12.9 expected) born lambs. This trend may simply be a function of late born lamb availability, as they were less likely to die of predation, or it could be a result of increased horizontal disease transmission. For example, lambs born early in the season would be present when sheep densities were at their lowest as most ewes had not given birth and remained on wintering grounds. Lambs born later in the year would arrive as sheep densities on the lambing grounds were at their highest. Assuming lamb immune systems are weakest during the first few weeks of life, late born lambs would have a much greater chance of coming into contact with other diseased animals, which could increase their chance of contracting the disease.

The sustained high levels of juvenile mortality we observed indicate these 3 populations are declining, primarily as a result of chronic pneumonia epizootics. Whether these pathogens are being maintained and transmitted among populations via bighorn sheep movements or from contact with domestic sheep and goats remains unclear. Over the course of our study we observed no range overlap between the Hill City and the other 2 subherds, and limited overlap during the lambing season between Rapid Creek and Spring Creek subherds (Figure 1). However, our sample of collared adults only included females, and it could be male movements, especially during the breeding season, could account for pathogen transmission. Conversely, bighorn sheep habitat in the Black Hills is made up of a matrix of public and private lands with several domestic sheep and goats present in areas adjacent to primary habitats or along known dispersal corridors. As effective buffers between domestics and bighorns have been identified as 20–40 km [47], [48], the potential exists for all 3 herds to have contact with domestic sheep and goats, precipitating pneumonia-caused mortality.

## Conclusions

We provide the first evaluation of the influence of intrinsic variables on neonate bighorn sheep survival and a quantitatively robust assessment of cause-specific mortality. Pneumonia was the major factor limiting recruitment followed by predation, although mortality from predation seemed to be partly compensatory to pneumonia and its effects were less pronounced as alternative prey became available. Given the politically untenable prospect of culling herds (J. Kanta, SDGF&P, personal communication), and the current lack of effective vaccines for wild bighorn sheep [16], [49], it seems current declines in these 3 populations will likely go unabated. Future research assessing the role of male dispersal in perpetuating disease among populations, experimenting with vaccines that have shown promise in captive bighorns at reducing pneumonia-caused mortality [50], and quantifying the relationship between disease and predation at limiting bighorn sheep populations is warranted. Furthermore, given the high rates of pneumonia-caused mortality we observed, and the apparent lack of pneumonia-causing pathogens in bighorn populations in the western Black Hills (B. Parr, South Dakota State University, Brookings, SD, unpublished data), management activities should be geared towards eliminating contact between diseased and healthy populations.
